# Association of ADHD symptoms with type 2 diabetes and cardiovascular comorbidities in adults receiving outpatient diabetes care

**DOI:** 10.1016/j.jcte.2023.100318

**Published:** 2023-04-11

**Authors:** Ali Zare Dehnavi, Yanli Zhang-James, Dan Draytsel, Ben Carguello, Stephen V. Faraone, Ruth S. Weinstock

**Affiliations:** a School of Medicine, Tehran University of Medical Sciences, Tehran, Iran; b Department of Psychiatry and Behavioral Sciences, SUNY Upstate Medical University, Syracuse, NY, USA; c Norton College of Medicine, SUNY Upstate Medical University, Syracuse, NY, USA; d Department of Biotechnology, SUNY ESF, Syracuse, NY, USA; e Department of Neuroscience and Physiology, SUNY Upstate Medical University, Syracuse, NY, USA; f Department of Endocrinology, Diabetes and Metabolism, SUNY Upstate Medical University, Syracuse, NY, USA; g Joslin Diabetes Center, SUNY Upstate Medical University, Syracuse, NY, USA

**Keywords:** Type 2 diabetes mellitus, Attention-deficit/hyperactivity disorder, Adult ADHD Self-Report Scale (ASRS), Cardiovascular disease

## Abstract

**Background:**

The relationship between attention-deficit/hyperactivity disorder (ADHD) symptoms and type 2 diabetes mellitus (T2D) and its cardiovascular outcomes have not been sufficiently studied.

**Methods:**

2,986 adults with T2D from the Joslin Diabetes Center at Upstate Medical University were assessed for ADHD-like symptoms, executive dysfunction, and emotional control using the Adult Self-Report Scale V1.1 (ASRS) expanded version. Surveys were sent electronically, and clinical data were obtained from the electronic medical record. Pearson chi-square test was used for categorical variables association. When ASRS scores were the dependent variable, negative binomial regression correcting for demographic variables that were associated with the ASRS scores was used.

**Results:**

155 (49.2%) of respondents met DSM-5 criteria for ADHD using the ASRS scores; Only ten (3.6%) of respondents had an ICD10 diagnosis of ADHD in their medical record; Forty-three (13.7%) had either a diagnosis of ADHD in the medical history or were taking medications used by people with ADHD. Higher levels of ADHD-like symptoms were found in patients with T2D compared with population norms. There was a modest association of the ASRS executive dysfunction subscale with overall cardiovascular comorbidities (p = 0.03). However, the p-value did not survive the multiple testing correction. Both ADHD-like symptoms and symptoms associated with emotional control, however, were not associated with specific cardiovascular diseases, hypertension, or with HbA1c, LDL-cholesterol, triglycerides, ALT, creatinine, or eGFR.

**Conclusion:**

Our results suggest that adults with T2D attending a tertiary care diabetes clinic are at risk for having ADHD-like symptoms, highlighting the importance of screening for ADHD symptoms in this specialty setting and referring undiagnosed adult patients for further assessment and treatment of ADHD. Larger studies are needed to clarify the relationship between ADHD-like symptoms, executive dysfunction, and emotional control with diabetic control and comorbidities.

## Highlights

• High levels of ADHD-like symptoms were observed in adults with type 2 diabetes (T2D) compared to population norms.
• 46% of T2D patients had ADHD-like symptoms, while under 14% had either a prior diagnosis or prescribed ADHD medications.
• No association was found between the presence of ADHD-like symptoms and HbA1c, hypertension, or dyslipidemia.

## Introduction

Attention-deficit/hyperactivity disorder (ADHD) is a neurodevelopmental disorder that affects between 2% and 7% of the population globally [1]. Symptoms of ADHD typically begin during early childhood and are characterized by impaired attention, increased impulsivity, and motor hyperactivity [2]. While the onset of symptoms typically occurs at a young age, symptoms often persist into adulthood [3]. Although psychiatric comorbidities among patients with ADHD have been very well documented [4], few large systematic studies had assessed the link between ADHD and somatic comorbidities [5]. Among the disorders with some evidence of association with ADHD is type 2 diabetes mellitus (T2D). T2D is associated with an increased risk of developing microvascular complications (e.g. neuropathy, nephropathy, and retinopathy) and macrovascular complications, having a high risk of developing cardiovascular diseases [6].

Several large population studies have examined the association between ADHD and T2D. A longitudinal study using the Taiwan National Health Insurance Research Database enrolled over 35,000 patients with ADHD and over 70,000 age- and sex-matched controls. Adolescents and young adults with ADHD were about three times more likely to develop T2D [7]. In a separate case-control study of 4,302 children and adolescents (age 5 to 15) with newly diagnosed ADHD from the same Taiwan database, those with ADHD were almost three times more likely to have had prior diagnoses of T2D, when compared with 21,510 randomly selected controls[8]. Another cohort study of 5,551,807 adults aged 18 to 64 years from multiple Swedish national registers found that the prevalence of T2D was 70% greater among adults with ADHD compared with those not diagnosed with ADHD [9]. Various shared underlying causes such as immune system dysfunction, oxidative stress, and behavioral factors have been reported to be responsible for the association of ADHD and metabolic diseases [10], [11]. A few studies on T2D patients have also documented a reduction of white matter volume, particularly in the frontal area that is responsible for attention, cognition, and motor functions [8], [12], [13]. Impairment of this area can induce inattention and impulsivity leading to a higher risk of ADHD [8]. Furthermore, several risk factors such as obesity and smoking that are linked with ADHD can cause insulin resistance and hyperglycemia [14], [15], [16], [17].

Some studies have shown that adults with T2D and ADHD have significantly higher levels of fasting blood glucose (FBG), postprandial blood glucose (PBG), hemoglobin A1C (HbA1c), and required insulin dose/kg than those without ADHD [18], [19], [20], [21]. It has also been reported that diagnosis of ADHD in adults with T2D is associated with an increased risk of diabetes-related complications such as neuropathy, ulcers, limb amputation, albuminuria, chronic renal failure, diabetic ketoacidosis, and elevated systolic blood pressure [18], [19]. Adults with T2D and ADHD have also been found to have more emergency room admissions, greater annual hospitalization rates, longer hospital stays, and higher annual health spending costs in comparison to those without ADHD [18], [22]. Although there is limited evidence suggesting an association between ADHD and T2D and its complications, little is known about how one disorder affects the symptomology and clinical outcomes of the other in people with dual diagnoses. It is particularly important to better understand the impact of ADHD on diabetes-associated cardiovascular comorbidities, given their high morbidity and mortality.

In the current study, we assessed ADHD-like symptoms among 312 adults with T2D and examined if their ADHD-like symptom counts or subscales of executive dysfunction and emotional control were associated with cardiovascular comorbidities, elevated HgbA1c (worse glycemic control), LDL-cholesterol and triglycerides (risk factors for cardiovascular disease), alanine transaminase (ALT; (a liver function test associated with fatty liver in T2D) or reduced kidney function (eGFR; estimated glomerular filtration rate).

## Methods

### Sample

Individuals with T2D (ICD-10-CM codes E11.XX), ages ≥ 18 years, receiving diabetes care at the Joslin Diabetes Center at Upstate Medical University in Syracuse NY from November 2019 to November 2021 with email addresses in the electronic medical record (EHR; Epic) were identified. They were contacted electronically and asked to complete surveys in RedCap. Consent for the completion of the surveys were obtained electronically. Consent to have additional data (described below) extracted from Epic were also obtained electronically. All data were deidentified. This work was approved by the Institutional Review Board for the Protection of Human Subjects of SUNY Upstate Medical University.

### Assessments

Participants were asked to complete the Adult Self-Report Scale, V1.1 (ASRS) expanded version, which assesses all 18 DSM-5 symptoms of ADHD (nine symptoms for inattention and nine for hyperactivity/impulsivity) and nine symptoms of executive dysfunction and four symptoms of emotional control. Each symptom is rated on a five-point scale indicating the frequency of the symptom over the past month. The scale’s categories are never, rarely, sometimes, often, and very often. Following Adler et al. [23], a symptom was scored as present if it occurred often or very often. The ASRS does not provide a diagnosis of ADHD because it does not assess impairment, the occurrence of symptoms in two or more settings and the age at onset of symptoms. However, as a screening tool ASRS has been validated to accurately detect the vast majority of general population cases at a threshold that also has high specificity and PPV (sensitivity, 91.4%; specificity, 96.0%; AUC, 0.94; PPV, 67.3%, [24]. We defined participants as ASRS Positive if they met DSM-5 symptom criteria for ADHD, which requires five or more inattention symptoms, or five or more hyperactivity/impulsivity symptoms [25].

From the electronic medical record Epic, the following data were extracted: age, sex, race/ethnicity, insurance type (Medicare, Medicaid, commercial, other, uninsured), body mass index (≥30 kg/m^2^ defined obesity), smoking status, and laboratory results for HbA1c, LDL-cholesterol, triglycerides, ALT, creatinine and estimated glomerular filtration rate (eGFR). From the problem list and medical history fields in Epic, the presence of several diagnoses was obtained including ADHD (ICD-10-CM codes F90.X), hypertension (I10-I15) and cardiovascular diseases (peripheral artery/vascular diseases, coronary artery disease, ischemic heart disease, subarachnoid bleeding, hemorrhagic stroke, cerebrovascular disease or ischemic stroke, deep vein thrombosis, pulmonary emboli, arrythmias including atrial fibrillation/flutter and supraventricular or ventricular tachycardia, cardiac arrest, heart failure, and venous thrombo-embolism). Medications used to treat ADHD (methylphenidate, amphetamine, guanfacine, clonidine, atomoxetine, viloxazine) and insulin use were obtained from medication lists in Epic.

### Statistical analysis

We used the Pearson chi-square test for categorical variables association. When ASRS scores were the dependent variable, negative binomial regression correcting for demographic variables that were associated with the ASRS scores were used. We chose negative binomial regression because the ASRS scores are count variables ranging from zero to 18, the total number of symptoms possible. An alpha level of 0.05 was used to assert statistical significance with multiple comparison corrections for our three primary outcomes which were tests of the association of any cardiovascular condition with each of the three ASRS scales.

## Results

Of the 2,986 individuals with T2D asked to complete the ASRS, 315 responded (10.5% response rate), of which 278 (89.1%) also consented to the extraction of data from the electronic medical record and 271 (88.3%) completed the survey fully; 155 (49.2%) of respondents met symptom diagnostic criteria for ADHD on the ASRS (“ASRS positive”); Ten (3.6%) of respondents had an ICD10 diagnosis of ADHD in their medical record; Forty-three (13.7%) had either a diagnosis of ADHD or were taking medications used by people with ADHD. Demographic features of study population in ASRS positive and ASRS negative groups are summarized in Table 1, along with the means and standard deviations (SD) of their ASRS symptom counts for the total DSM-5 ADHD symptoms and the subcategories.

**Table 1:** Demographic characteristics and symptom numbers in ASRS positive and ASRS negative groups.

**Group (n = 315)**	**ASRS Positive** **(%; n = 155)**	**ASRS Negative** **(%; n = 160)**	**Statistics**
Age (mean ± SD)	59.6 ± 13.6	65.7 ± 12.2	z = -3.7, p < 0.001
Female (No,%)	73 (57.9%)	66 (43.4%)	Χ^2^ = 5.81, p = 0.016
Caucasian (n = 243)	72.9	81.2	Χ^2^ = 13.6, p = 0.001
African American (n = 21)	3.9	9.4	
Other (n = 51)	23.2	9.4	
Hispanic (n = 40)	20.0	5.6	Χ^2^ = 16.1, p = < 0.001
Non-Hispanic (n = 271)	78.0	93.8	
Other (n = 4)	2.0	0.6	
**ASRS Symptom Counts (Mean ± SD)**			
Total ADHD Symptoms	13.7 ± 3.5	3.9 ± 2.9	
Inattention	7.7 ± 1.5	2.1 ± 1.7	
Hyperactivity/Impulsivity	6.1 ± 2.6	1.8 ± 1.6	
Emotional Dyscontrol	3.1 ± 1.2	1.2 ± 1.2	
Executive Dysfunction	5.2 ± 2.0	1.5 ± 1.6	

As shown in Table1, ASRS positive participants were significantly less likely to be Caucasian and more likely to be Hispanic compared with ASRS negative participants. In the ASRS positive cohort, 57.9% were female and 42.1% were male. For the ASRS negative cohort, these rates were 43.4% and 56.6 %, respectively (Χ^2^ = 5.81, p = 0.016). For the ASRS positive compared to the ASRS negative cohort, the mean (SD) ages were 59.6 (13.6) years vs 65.7 (12.2) years respectively (z = -3.7, p < 0.001).

Table2 compares T2D adults with and without any cardiovascular disease (CVD) on the number of ADHD, executive dysfunction, and emotional dyscontrol symptoms reported on the ASRS. The two groups did not differ significantly in the total number of ADHD or emotional dyscontrol symptoms. We did, however, find modest evidence that the groups differed in executive dysfunction symptoms (p = 0.03, which exceeds p < 0.017, the p-value threshold appropriate for testing three primary outcomes). Because of these possible differences in executive dysfunction, we examined individual CVDs to determine if any individual cardiovascular disease diagnosis was associated with executive dysfunction. As Table 3 shows, none of these diseases showed a statistically significant association with the presence of executive dysfunction symptoms, but these analyses are limited by the relatively few numbers of individuals with each condition.

**Table 2:** Number of ADHD, Executive Dysfunction and Emotional Dyscontrol Symptoms in T2D Adults with and without Cardiovascular Disease.

	Any CVD (Mean, SD)	No CVD (Mean, SD)	z-statistic	p-value
Type of Symptoms:	N = 36	N = 242		
ADHD n = 315	7.9(5.4)	8.8(5.6)	0.78	0.44
Executive Dysfunction n = 315	3.1(2.5)	2.8(2.4)	2.23	0.03
Emotional Dyscontrol n = 315	2.0(1.5)	2.3(1.4)	0.50	0.62

Note: CVD = Any cardiovascular disease; ASRS includes 18 ADHD symptoms, nine executive dysfunction symptoms and four emotional control symptoms.

**Table 3:** Association of Executive Dysfunction with Individual Cardiovascular Conditions.

	Z Value	P Value
Hypertension	0.73	0.46
Peripheral Artery Disease	0.29	0.77
Acute Myocardial Infarction	0.72	0.47
Acute Coronary Syndrome	0.73	0.47
Any Ischemic Heart Disease	−0.20	0.84
Subarachnoidal Bleeding	−0.06	0.96
Hemorrhagic Stroke	−0.60	0.55
Ischemic Stroke	−0.23	0.82
Deep Vein Thrombosis	−0.03	0.98
Pulmonary Emboli	1.14	0.25
Atrial Fibrillation Flutter	0.32	0.75
Supraventricular Tachycardia	1.61	0.11
Ventricular Tachycardia	1.39	0.16
Cardiac Arrest	0.73	0.46
Peripheral Vascular Diseases Arteriosclerosis	1.17	0.24
Arrhythmia Broad	1.58	0.11
Heart Disease Heart Failure	0.25	0.80
Cerebrovascular Disease Ischemic Stroke	−0.96	0.34

The numbers of ADHD-like symptoms, executive dysfunction symptoms, and emotional control symptoms were also not significantly associated with the individual’s most recent laboratory values of HbA1c, LDL-cholesterol, triglycerides, ALT, creatinine, or eGFR (all p's > 0.08; data not shown).

In exploratory analyses, we found no significant association between the EHR diagnoses of ADHD, total ASRS symptoms or total emotional control symptoms with any of the individual CVDs in Table 3 (all p's > 0.10). EHR diagnoses of ADHD, and the numbers of ADHD-like symptoms, executive dysfunction symptoms and emotional control symptoms were not significantly associated with the participants most recent laboratory values of HbA1c, LDL-cholesterol, triglycerides, ALT, creatinine, or eGFR (all p's > 0.08; data not shown).

**Fig. 1:**
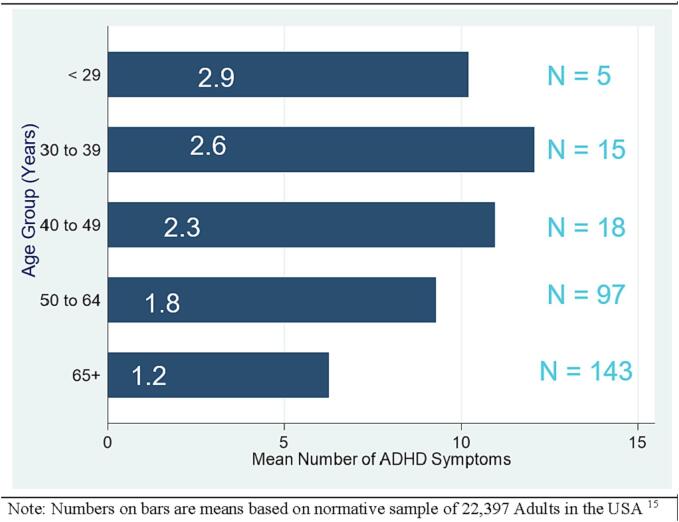
Association of Age and ADHD Symptoms in Adults with Type 2 Diabetes.

The number of ASRS reported symptoms of ADHD declined significantly with age (z = -4.8, p < 0.001), Fig. 1 shows this decline stratified by age. These data were compared to normative data based on responses from 22,397 adults in the US population [26]. As the Figure shows, the mean number of symptoms reported by T2D adults is much higher than the means reported in the normative sample. For all age strata, the normative value was outside of the 95% confidence interval for the mean number of ADHD-like symptoms in our sample. Our sample sizes are small for the three youngest age groups so those findings should be viewed cautiously.

## Discussion

In this first study of the presence of symptoms of ADHD and cardiovascular diseases among adults with T2D, we found evidence for high levels of ADHD-like symptoms compared with population norms. We also find a modest association of ADHD executive dysfunction subscale with the overall cardiovascular comorbidity status, although the p-value did not survive multiple correction. The ADHD-like symptoms, as well as the symptoms of emotional dysregulation, however, were not associated with specific cardiovascular diseases or with laboratory values of HbA1c, LDL-cholesterol, triglycerides, ALT, creatinine, or eGFR.

The prevalence of an ADHD diagnosis in people with diabetes has been reported to be variable, ranging from about 2% to almost 12% in different studies [19], [22], [27], [28]. The difference in the ADHD diagnosis rate may be explained by the variation in diagnostic criteria and also the clinical characteristics of the studies’ population [29]. In our study, approximately 46% of the study population met symptom diagnostic criteria for ADHD based on the ASRS. In contrast, <4% of them had a prior diagnosis of ADHD and<14% had either a prior diagnosis of ADHD or a prescription history of ADHD medications in their medical records. Despite the limitation that some of the ADHD medications that we included in our analysis could also be used for other conditions such as anxiety, depression, and hypertension, the total percentage of diagnosed or treated ADHD patients was still well below the ASRS positive rates. This difference highlights the high prevalence of undiagnosed and untreated ADHD symptoms among T2D adults. It is also possible that adults with ADHD were more likely to have participated in our study. Thus, primary care providers and endocrinologists should be aware of the need to screen adults with diabetes for ADHD.

Undiagnosed ADHD in people with diabetes has been shown to have a negative impact on diabetes treatment and metabolic control [30]. A study by Nylander et al. revealed that adolescents with undiagnosed ADHD and diabetes were mostly male and had elevated HbA1C levels [30]. Studies that have used behavioral scales such as Five-To-Fifteen or the Child Behavior Checklist (CBCL) also found that ADHD symptoms were positively associated with high HbA1c levels in patients with T1D (>70 mmol/mol or more, [31], [32], indicative of poor metabolic control in patients with more ADHD symptoms. Furthermore, youth with T1D and untreated ADHD had worse HbA1c and more hospitalizations than those with dual diagnoses but treated with ADHD pharmacotherapies [33]. Despite the fact that most of these studies have been conducted in children and adolescents with T1D, and ADHD symptoms may be different in adults, findings do support that managing ADHD symptoms can help improve diabetes management in adults too [34]. HbA1c levels are also higher in children, adolescents, and young adults with type 1 diabetes compared to middle-aged and metabolic control..older adults [35]. The impact of ADHD on glycemic control in adults with T2D requires further study.

Among ADHD characteristics, executive functioning skills have been well documented to be associated with diabetes control and treatment adherence [32], [36], [37]. Cognitive decline including executive dysfunction is also associated with a longer duration of diabetes, a history of severe hypoglycemic episodes, and poor glycemic control [13], [38], [39]. Since cognitive impairment can adversely affect the ability of a person with diabetes to self-manage their disease, the American Diabetes Association recommends routine screening of cognition in adults ages 65 and older [40]. Our results suggest that for adults with diabetes, the presence of undiagnosed ADHD, particularly executive dysfunction, should be considered regardless of age.

In contrast with our findings, several previous studies have shown that the co-occurrence of ADHD in people with diabetes is associated with a greater rate of diabetes complications including, chronic renal failure [18], [22], [33]. A number of papers have reported that the dual diagnosis of ADHD and type 1 diabetes is associated with higher levels of blood glucose, HbA1c, higher systolic blood pressure and an increased number of diabetes-related complications including nephropathy [18], [19], [22]. The relatively few individuals in our study with advanced renal disease limited our ability to examine this association. Larger studies in adults with T2D are needed.

Cardiovascular comorbidities and complications are common in adults with T2D and are a leading cause of morbidity and mortality [41], [42]. Demographic, clinical, and laboratory risk factors including age, obesity, smoking history, hypertension, renal insufficiency, abnormal lipid profiles and elevated HbA1c are linked with the occurrence of CVD and CVD death in T2D [43], [44]. Low socioeconomic status, decreased social support, anger, anxiety, and depression have also been reported to be associated with higher rates of CVD [45], [46]. Anxiety, according to a *meta*-analysis of over 2 million people, significantly increases the risk of CVD mortality and is linked to an increased risk of coronary heart disease, stroke, and heart failure [47]. Limited publications have also examined heart disease in ADHD, but they have mainly focused on the effects of ADHD medications on the risk of CVD, and the role of ADHD symptoms has been rarely examined [48], [49]. Our findings suggest that there is a need to further investigate the impact of ADHD symptoms in adults on the development of CVD.

There are several limitations to this study. Our survey response rate was 10.4% (n = 312) and it is possible that T2D adults with ADHD preferentially responded. In-person interview using the ASRS screening questionnaire may help to decrease information bias. A comparison group (another chronic medical disease) would have been useful to control for nonspecific effects. Future studies with larger sample sizes may help to further clarify the question regarding how ADHD symptoms, executive dysfunction and emotional control may affect metabolic control and CVD comorbidities. Although EHR data are widely used for research, diagnostic codes are not uniformly applied across providers. However, a strength of our study includes investigating the association of numerous different cardiovascular conditions with ADHD-like symptoms in adults with T2D.

In conclusion, our results suggest that adults with T2D attending a tertiary care diabetes center are at high risk for having symptoms of ADHD. Given that the symptoms of ADHD can interfere with self-care and diabetes management, clinicians treating adults with T2D should consider screening for ADHD.

## Funding

The project has received funding from the European Union’s Horizon 2020 research and innovation programme under grant agreement No 965381. This report reflects only the author’s view, and the European Union is not responsible for any use that may be made of the information it contains.

## Conflict of interest

In the past year, Dr. Faraone received income, potential income, travel expenses continuing education support and/or research support from Aardvark, Aardwolf, Tris, Otsuka, Ironshore, KemPharm/Corium, Akili, Supernus, Atentiv, Noven, Axsome and Genomind. With his institution, he has US patent US20130217707 A1 for the use of sodium-hydrogen exchange inhibitors in the treatment of ADHD. He also receives royalties from books published by Guilford Press: Straight Talk about Your Child’s Mental Health, Oxford University Press: Schizophrenia: The Facts and Elsevier: ADHD: Non-Pharmacologic Interventions. He is Program Director of www.ADHDEvidence.org and www.ADHDinAdults.com.

Dr Weinstock participates in multicenter clinical trials, through her institution, sponsored by Eli Lilly, Novo Nordisk, Medtronic, Insulet, Kowa and Boehringer Ingelheim. Tandem has donated insulin pumps and DexCom continuous glucose monitors for clinical studies. She also receives royalties for her contributions to Up-to-Date.

All other authors declare no conflict of interest.

## CRediT authorship contribution statement

**Ali Zare Dehnavi:** . **Yanli Zhang-James:** Supervision. **Dan Draytsel:** Data curation. **Ben Carguello:** Data curation. **Stephen V. Faraone:** Conceptualization. **Ruth S. Weinstock:** Conceptualization, Data curation, Supervision.

## Declaration of Competing Interest

The authors declare that they have no known competing financial interests or personal relationships that could have appeared to influence the work reported in this paper.

## References

[1] Sayal K. (2018). ADHD in children and young people: prevalence, care pathways, and service provision. Lancet Psychiatry.

[2] Landau Z., Pinhas-Hamiel O. (2019). Attention Deficit/Hyperactivity, the Metabolic Syndrome, and Type 2 Diabetes. Curr Diab Rep.

[3] Chen M.H. (2013). Attention deficit hyperactivity disorder, tic disorder, and allergy: is there a link? A nationwide population-based study. J Child Psychol Psychiatry.

[4] Faraone S.V. (2021). The World Federation of ADHD International Consensus Statement: 208 Evidence-based Conclusions about the Disorder. Neurosci Biobehav Rev.

[5] Cortese S. (2016). Association Between ADHD and Obesity: A Systematic Review and Meta-Analysis. Am J Psychiatry.

[6] Cho N.H. (2018). IDF Diabetes Atlas: Global estimates of diabetes prevalence for 2017 and projections for 2045. Diabetes Res Clin Pract.

[7] Chen, M.H., et al., Risk of Type 2 Diabetes in Adolescents and Young Adults With Attention-Deficit/Hyperactivity Disorder: A Nationwide Longitudinal Study. J Clin Psychiatry, 2018. **79**(3): p. 17m11607. 10.4088/JCP.17m11607 29727071

[8] Chen H.J. (2013). Association of attention-deficit/hyperactivity disorder with diabetes: a population-based study. Pediatr Res.

[9] Chen Q. (2018). Common psychiatric and metabolic comorbidity of adult attention-deficit/hyperactivity disorder: A population-based cross-sectional study. PLoS One.

[10] Joseph N. (2015). Oxidative Stress and ADHD: A Meta-Analysis. J Atten Disord.

[11] Nielsen P.R., Benros M.E., Dalsgaard S. (2017). Associations between autoimmune diseases and attention-deficit/hyperactivity disorder: a nationwide study. J Am Acad Child Adolesc Psychiatry.

[12] Hsu J.-L. (2012). Microstructural white matter abnormalities in type 2 diabetes mellitus: a diffusion tensor imaging study. Neuroimage.

[13] Rawlings A.M. (2014). Diabetes in midlife and cognitive change over 20 years: a cohort study. Ann Intern Med.

[14] McClernon F.J., Kollins S.H. (2008). ADHD and smoking: from genes to brain to behavior. Ann N Y Acad Sci.

[15] Chen Q. (2017). Shared familial risk factors between attention-deficit/hyperactivity disorder and overweight/obesity–a population-based familial coaggregation study in Sweden. J Child Psychol Psychiatry.

[16] Willi C. (2007). Active smoking and the risk of type 2 diabetes: a systematic review and meta-analysis. JAMA.

[17] Guilherme A. (2008). Adipocyte dysfunctions linking obesity to insulin resistance and type 2 diabetes. Nat Rev Mol Cell Biol.

[18] Vinker-Shuster M. (2022). Glycemic Control and Diabetes Related Complications in Adults with Type 1 Diabetes Mellitus and ADHD. J Atten Disord.

[19] Hilgard D. (2017). Comorbidity of attention deficit hyperactivity disorder and type 1 diabetes in children and adolescents: Analysis based on the multicentre DPV registry. Pediatr Diabetes.

[20] Yazar A. (2019). The effect of attention deficit/hyperactivity disorder and other psychiatric disorders on the treatment of pediatric diabetes mellitus. Pediatr Diabetes.

[21] Khan, S., et al., PMH49 assessment of type 2 diabetes mellitus patients with and without symptoms of ADHD: Patient characteristics and resource utilization data from an internet-based survey. Value in Health, 2009. **3**(12): p. A181-A182.

[22] Vinker-Shuster M. (2019). Attention-Deficit Hyperactivity Disorder in Pediatric Patients With Type 1 Diabetes Mellitus: Clinical Outcomes and Diabetes Control. J Dev Behav Pediatr.

[23] Green J.G. (2019). Evidence for the reliability and preliminary validity of the Adult ADHD Self-Report Scale v1.1 (ASRS v1.1) Screener in an adolescent community sample. Int J Methods Psychiatr Res.

[24] Ustun B. (2017). The World Health Organization Adult Attention-Deficit/Hyperactivity Disorder Self-Report Screening Scale for DSM-5. JAMA Psychiat.

[25] American Psychiatric Association, *Diagnostic and statistical manual of mental disorders (5th ed.)*. 5th ed, ed. A.P. Association. 2013, Arlington, VA: American Psychiatric Publishing.

[26] Adler L.A. (2019). Establishing US norms for the Adult ADHD Self-Report Scale (ASRS-v1.1) and characterising symptom burden among adults with self-reported ADHD. Int J Clin Pract.

[27] Kapellen T.M. (2016). Prevalence of medically treated children with ADHD and type 1 diabetes in Germany - Analysis of two representative databases. J Pediatr Endocrinol Metab.

[28] Macek J. (2019). Impact of attention deficit hyperactivity disorder on metabolic control in adolescents with type1 diabetes. J Psychosom Res.

[29] Cabral, M.D.I., S. Liu, and N. Soares, *Attention-deficit/hyperactivity disorder: diagnostic criteria, epidemiology, risk factors and evaluation in youth.* Transl Pediatr, 2020. **9**(Suppl 1): p. S104-s113. 10.21037/tp.2019.09.08 PMC7082246 32206588

[30] Nylander C. (2018). Previously undiagnosed attention-deficit/hyperactivity disorder associated with poor metabolic control in adolescents with type 1 diabetes. Pediatr Diabetes.

[31] Nylander C. (2013). Children and adolescents with type 1 diabetes and high HbA1c – a neurodevelopmental perspective. Acta Paediatr.

[32] Nylander C. (2018). Self- and parent-reported executive problems in adolescents with type 1 diabetes are associated with poor metabolic control and low physical activity. Pediatr Diabetes.

[33] Mazor-Aronovitch K. (2021). Dual diagnosis of type 1 diabetes mellitus and attention deficit hyperactivity disorder. Pediatr Diabetes.

[34] Ramtekkar, U.P., et al., Sex and age differences in attention-deficit/hyperactivity disorder symptoms and diagnoses: implications for DSM-V and ICD-11. J Am Acad Child Adolesc Psychiatry, 2010. **49**(3): p. 217-28.e1-3. PMC3101894 20410711

[35] Miller K.M. (2015). Current state of type 1 diabetes treatment in the U.S.: updated data from the T1D Exchange clinic registry. Diabetes Care.

[36] McNally K. (2010). Executive functioning, treatment adherence, and glycemic control in children with type 1 diabetes. Diabetes Care.

[37] Nunley K.A. (2015). Clinically Relevant Cognitive Impairment in Middle-Aged Adults With Childhood-Onset Type 1 Diabetes. Diabetes Care.

[38] Lee A.K. (2018). Severe hypoglycaemia, mild cognitive impairment, dementia and brain volumes in older adults with type 2 diabetes: the Atherosclerosis Risk in Communities (ARIC) cohort study. Diabetologia.

[39] Yaffe K. (2012). Diabetes, glucose control, and 9-year cognitive decline among older adults without dementia. Arch Neurol.

[40] American Diabetes Association Professional Practice Committee, *13. Older Adults: Standards of Medical Care in Diabetes—2022.* Diabetes Care, 2021. **45**(Supplement_1): p. S195-S207. 10.2337/dc22-S013 PMC8935395 34964847

[41] Einarson T.R. (2018). Prevalence of cardiovascular disease in type 2 diabetes: a systematic literature review of scientific evidence from across the world in 2007–2017. Cardiovasc Diabetol.

[42] Ma C.X. (2022). Cardiovascular disease in type 2 diabetes mellitus: progress toward personalized management. Cardiovasc Diabetol.

[43] Tamiru S., Alemseged F. (2010). Risk Factors for Cardiovascular Diseases among Diabetic Patients In Southwest Ethiopia. Ethiop J Health Sci.

[44] Fuller J.H., Stevens L.K., Wang S.L. (2001). Risk factors for cardiovascular mortality and morbidity: the WHO Mutinational Study of Vascular Disease in Diabetes. Diabetologia.

[45] Albus C. (2010). Psychological and social factors in coronary heart disease. Ann Med.

[46] Suls J., Bunde J. (2005). Anger, anxiety, and depression as risk factors for cardiovascular disease: the problems and implications of overlapping affective dispositions. Psychol Bull.

[47] Emdin C.A. (2016). Meta-Analysis of Anxiety as a Risk Factor for Cardiovascular Disease. Am J Cardiol.

[48] Liu H., Feng W., Zhang D. (2019). Association of ADHD medications with the risk of cardiovascular diseases: a meta-analysis. Eur Child Adolesc Psychiatry.

[49] Olfson, M., et al., *Stimulants and cardiovascular events in youth with attention-deficit/hyperactivity disorder.* J Am Acad Child Adolesc Psychiatry, 2012. **51**(2): p. 147-56. 10.1016/j.jaac.2011.11.008 PMC3266532 22265361

